# Effects of lutein supplementation in age-related macular degeneration

**DOI:** 10.1371/journal.pone.0227048

**Published:** 2019-12-30

**Authors:** Liwen Feng, Kailai Nie, Hui Jiang, Wei Fan

**Affiliations:** 1 Department of Ophthalmology, West China Hospital of Sichuan University, Chengdu, Sichuan Province, China; 2 Research Laboratory of Ophthalmology and Vision Sciences, State Key Laboratory of Biotherapy, West China Hospital, Sichuan University, Chengdu, China; Indiana University School of Medicine, UNITED STATES

## Abstract

The purpose of this meta-analysis was to evaluate the effects of lutein supplementation on macular pigment optical density (MPOD) in randomized controlled trials involving patients with age-related macular degeneration (AMD). A comprehensive search of the literature was performed in PubMed, Cochrane Library, Web of Science, China National Knowledge Infrastructure, Chinese Biomedical Literature Database, and Wan Fang database through December 2018. Nine randomized controlled trials involving 920 eyes (855 with AMD) were included. Meta-analysis suggested that lutein supplementation (10 or 20 mg per day) was associated with an increase in MPOD (mean difference (MD) 0.07; 95% confidence interval (CI) 0.03 to 0.10), visual acuity (MD 0.28; 95%CI 0.06 to 0.50) and contrast sensitivity (MD 0.26; 95%CI 0.22 to 0.30). Stratified analyses showed the increase in MPOD to be faster and greater with higher dose and longer treatment. The available evidence suggests that dietary lutein may be beneficial to AMD patients and the higher dose could make MPOD increase in a shorter time.

## Introduction

The macula in humans is a yellow pigmented area at the posterior of the eye that allows central vision and provides the most acute visual acuity and best color identification [1]. Macular yellow pigment was first described in 1945 as a carotenoid member of the leaf xanthophyll family [2], and more recently it was found to be primarily lutein and its structural isomer zeaxanthin [3]. Lutein is the most abundant carotenoid in the eye and brain [4]; its concentration is higher in the retina than in other tissues, and about 1,000 times higher in the retina than in serum [5]. Lutein is most dense at the macula, and its concentration declines rapidly in the peripheral regions. The concentration of lutein is approximately 2.5 times higher in the macula than in the peripheral retina. Macular pigments (MPs) concentrate in the photoreceptor axons of Henle nerve fiber layer and the rod outer segments [6], where they easily undergo oxidative attack. These carotenoids have been shown to play a key role in maintaining macular morphology and function [7, 8]. Lutein in the macula filters blue light, quenches free radicals and supports vision. As a result, lutein may play a role in prevention of age-related eye diseases, such as age-related macular degeneration and age-related cataract [9].

The levels of macular pigments, usually measured in terms of the macular pigment optical density (MPOD), can reflect retinal health status [7, 10–13]. Studies have shown significant correlations between high lutein concentration in ocular tissues or in serum and reduced risk of age-related macular degeneration (AMD) [14–16]. AMD is a leading cause of blindness in adults over 65 years old [17]. While its etiology is unclear, the disease seems to be related to excessive exposure to reactive oxygen species [18]. At present, no effective therapy exists for non-exudative AMD [1].

Since lutein must be obtained from the diet, principally from leafy green vegetables, fruits, and egg yolk [19], researchers have explored whether dietary supplementation with lutein might prevent AMD in healthy people or improve the condition of patients with AMD. Several studies support these possibilities [15, 20, 21] and have demonstrated that lutein supplementation can improve visual acuity [22]. On the other hand, one study showed that supplementation did not significantly improve MPOD in patients with early AMD [23]. One study showed no significant MPOD differences between healthy eyes and eyes with early AMD[13], although the results suggested that low MPOD is indeed related to visual function. Still other studies have concluded that melanin, rather than macular pigments, may protect against AMD [24, 25].

To help clarify these inconsistencies in the literature, we meta-analyzed available research on lutein supplementation in patients with AMD. We focused only on randomized controlled trials that measured MPOD in AMD patients who received dietary lutein or not.

## Methods

This meta-analysis was conducted in accordance with the Preferred Reporting Items for Systematic Reviews and Meta-analysis (PRISMA) guidelines [26]. And the review was registered on PROSPERO at the Centre for Reviews and Dissemination (CRD42019129281).

### Search strategy

A comprehensive search was performed in the following six electronic databases without data or language restriction through December 2018: PubMed, Cochrane Library, Web of Science, China National Knowledge Infrastructure, Chinese Biomedical Literature Database, and Wan Fang database. The detailed searching strategy for PubMed was as follows: (randomized controlled trial[Title/Abstract] OR randomized[Title/Abstract] OR placebo[Title/Abstract]) AND (AMD[Title/Abstract] OR age-related macular degeneration[Title/Abstract]) and (Carotenoids[Title/Abstract] OR Abscisic Acid[Title/Abstract] OR Retinoids[Title/Abstract] OR beta Carotene[Title/Abstract] OR zeta Carotene[Title/Abstract] OR Lutein[Title/Abstract] OR Canthaxanthin OR Norisoprenoids[Title/Abstract]). Additional studies were retrieved by hand from references in screened papers and systematic reviews identified during the searches.

### Study selection

To be included in this meta-analysis, studies had to involve (a) a randomized controlled design in which subjects were randomly assigned to treatment (supplementation) or placebo groups, (b) subjects who were diagnosed with AMD, (c) supplementation with lutein alone or lutein with other antioxidants, and (d) MPOD as an outcome. Literature searches were screened independently by two investigators after removing duplicates. Studies were screened first based on titles and abstracts, and the remaining studies were read in full and included if they fulfilled the inclusion criteria. Disagreements were resolved by discussion between the two investigators.

### Data extraction

The following data were extracted independently by two reviewers: first author, year of publication, country, sample size, age, gender, intervention, follow-up times and outcome measures. Primary outcomes, including MPOD, visual acuity, and concentration of lutein, were extracted for different time points and lutein doses. To eliminate the influence of different equipment used to measure MPOD across studies, we calculated the change in MPOD by subtracting the values before supplementation from the values after supplementation. Disagreements were resolved by consensus or opinion from a third reviewer.

### Study quality assessment

Risk of bias in each study was evaluated by two reviewers using the Cochrane Collaboration tool [27] which consists of the following six items: (a) random sequence generation; (b) allocation concealment; (c) blinding of participants and personnel; (d) blinding of outcome assessment; (e) incomplete outcome data; and (f) selective reporting. Disagreements were resolved by discussion between two reviewers.

### Statistical analysis

Data were pooled by Review Manager 5.3 software (Cochrane Collaboration 2014, Copenhagen, Denmark) and analyzed with random- or fixed-effects models. Data extracted were continuous and were therefore summarized as mean differences (MDs) with 95% confidence intervals (CIs) and compared between groups. Subgroup analyses were also performed to compare the effects of different doses and treatment durations. Statistical heterogeneity among studies was assessed using Q tests; the degree of heterogeneity, using the I^2^ value. Sensitivity analysis was conducted by excluding one study at a time and assessing whether the pooled results from the remaining studies differed from the results obtained across all studies. Publication bias was assessed using the Egger’s test in Stata software, version 13.1 (Stata Corp, College Station, TX, USA) and using a funnel plot generated by Review Manager 5.3 software. Admittedly, the funnel plot and Egger’s test is less reliable for the small number of studies in this meta-analysis [28].

## Results

### Studies included

**Fig 1:**
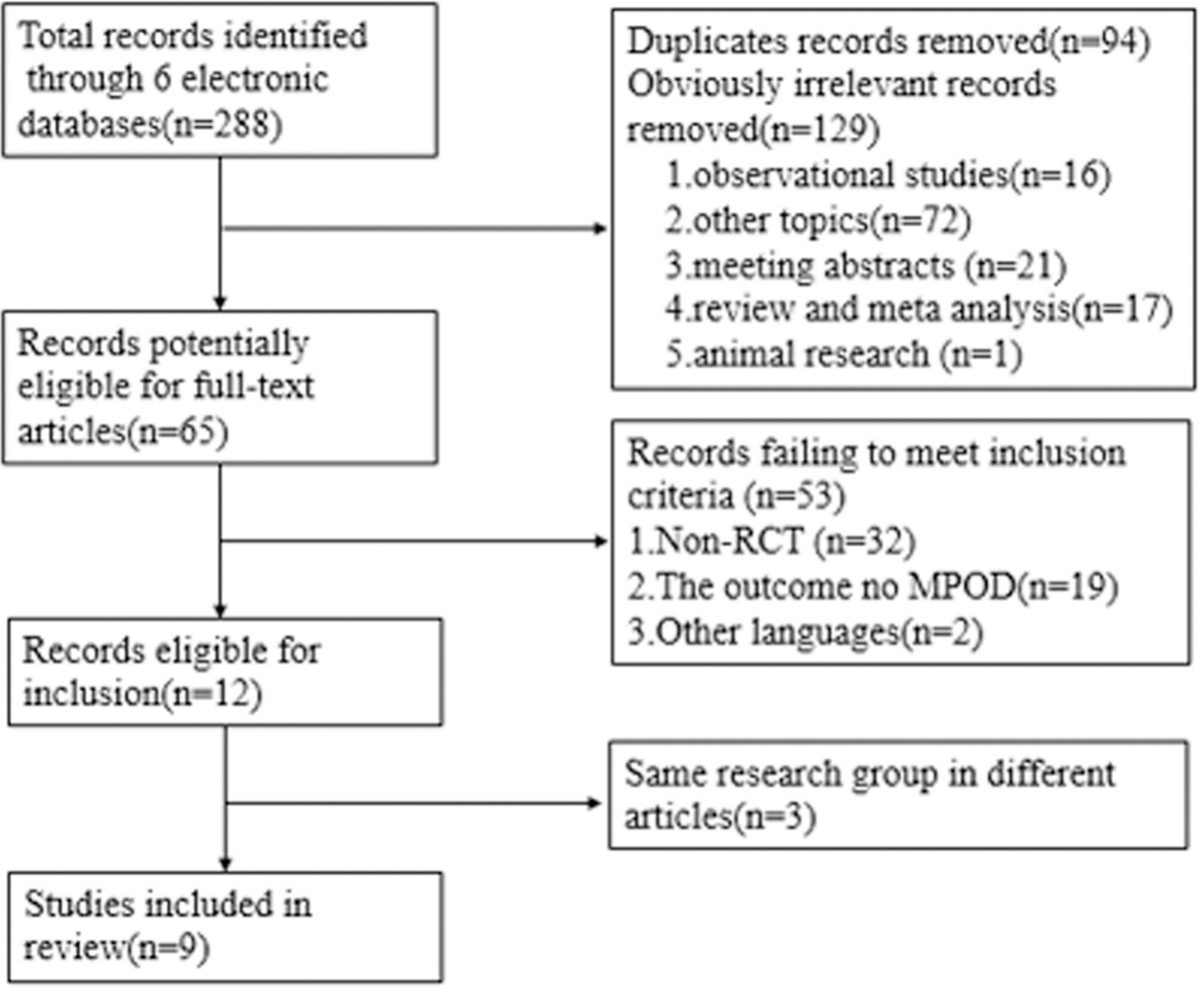
Flow diagram showing study inclusion and exclusion.

We obtained 288 relevant articles from our searches of six electronic databases through December 2018. After removing duplicates, case reports, reviews and meeting abstracts, the remaining 65 articles were read in full. After excluding 56 for failing to satisfy the inclusion criteria, nine studies were ultimately included in the meta-analysis (**Fig 1**).

### Characteristics of included studies

**Fig 2:**
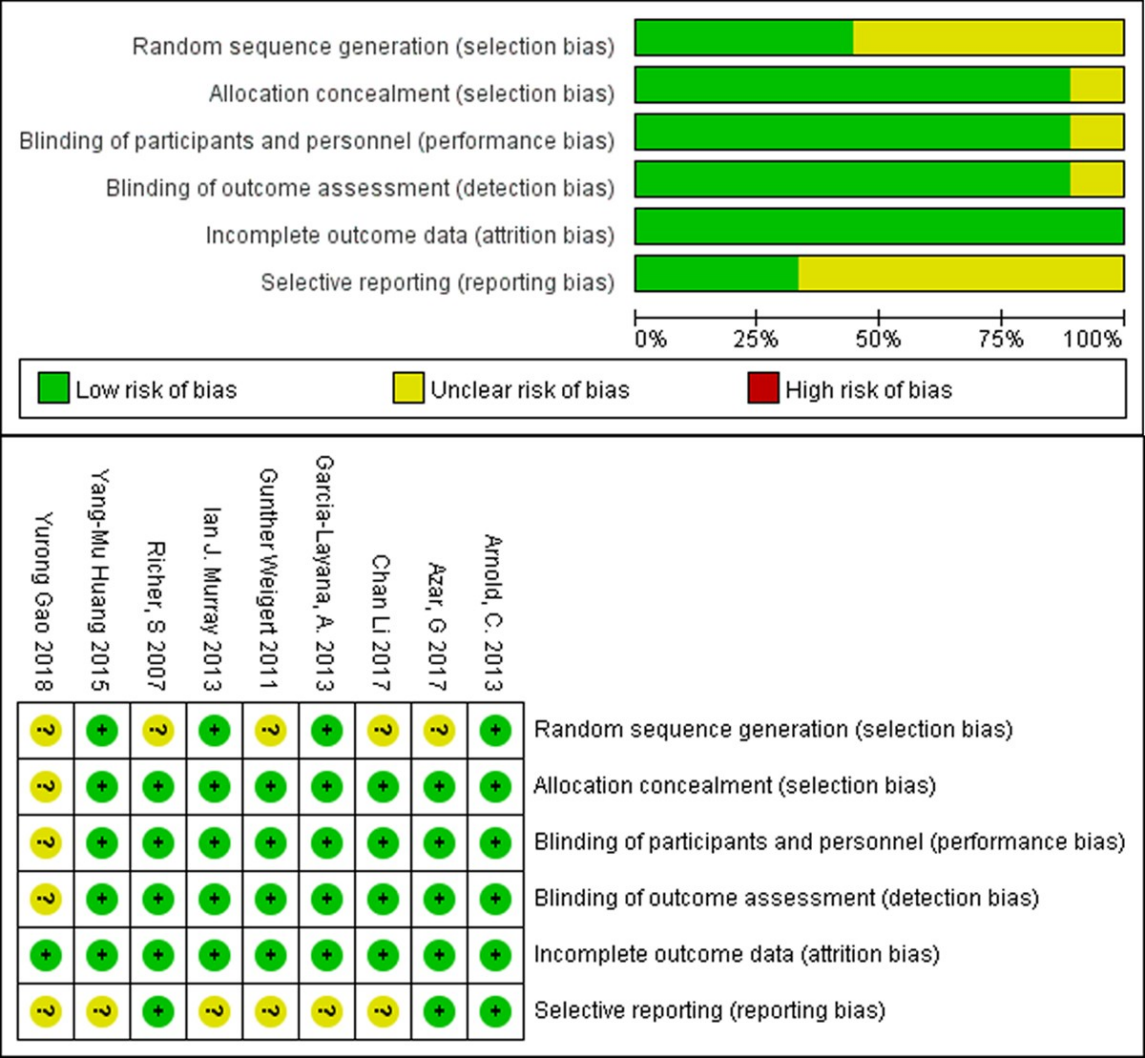
Risk of various biases and summary of the risk of bias, as determined by the Cochrane Collaboration tool.

**Fig 3:**
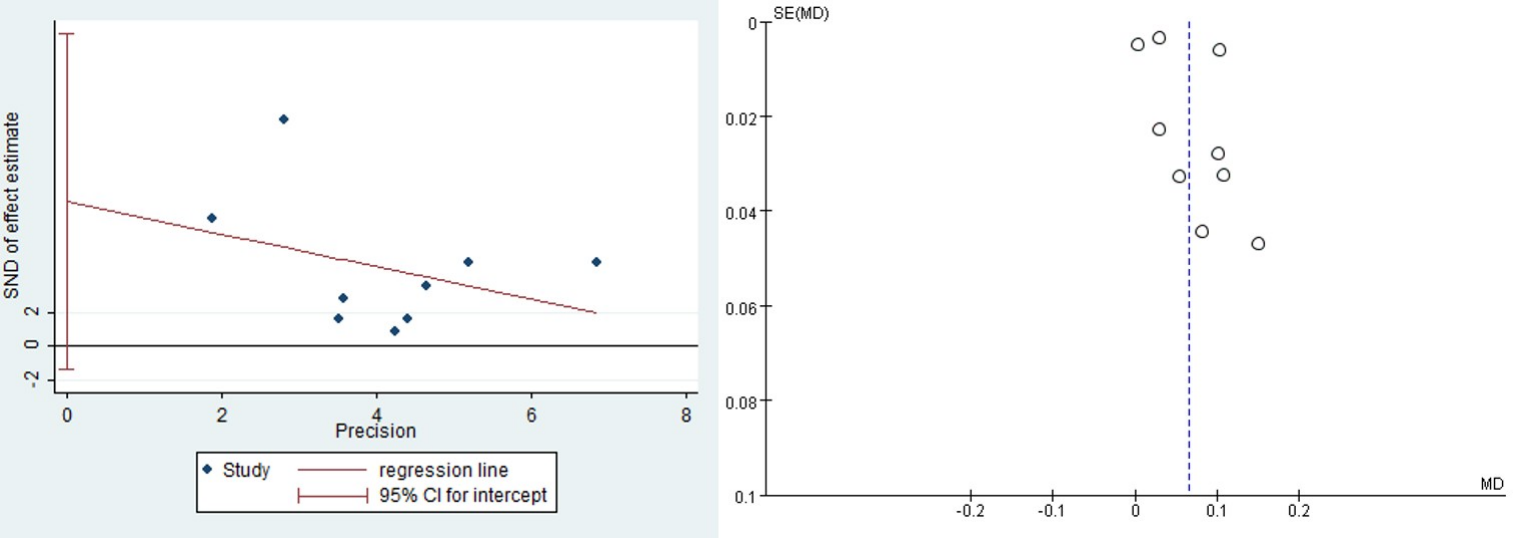
Egger’s test and funnel plot for MPOD across all studies.

Altogether, the nine prospective, randomized, controlled trials involved 855 patients (920 eyes) histologically diagnosed with AMD (**Table 1**). Patients in the treatment group received dietary lutein supplementation and supplementation in five studies also included other antioxidants. Patients in the control group received placebo. Four studies were conducted in Asia (3 in China, 1 in Lebanon), 3 in Europe, 1 in the US, and 1 in Australia. Follow-up periods were from 3 months to 2 years. All studies included MPOD as outcome; six also measured visual acuity, three reported lutein serum concentration, and two analyzed contrast sensitivity. Risk of bias was low in all studies, which were scored as high-quality (**Fig 2**). In sensitivity analysis, excluding any one of studies from the analysis did not change the overall results. Publication bias was not significant, based on Egger’s test (p = 0.081), and the funnel plot was symmetric (**Fig 3**).

**Table 1:** Characteristics of included randomized controlled trials.

Study	Participants	Participants’ characteristics	Interventions	Measurement Method for MPOD	Trial Duration
Yurong Gao et al. (2018)[29]	48 AMD patients; 52 eyes	24 dry AMD patients;24 wet AMD patients F 20; M 28 age 61.9±11.24	1.20mg/d lutein 2.blank	Fundus autofluorescence	3 months
Chan Li et al.(2017)[30]	200 early AMD patients	F101; M 99 age 70.22±7.13	1.20mg/d lutein 2.placebo	not given	12 months
Richer, S. P. et al.(2011)[31, 32]	60 mild-to-moderate AMD patients	F 3; M 57 age 74.9±10	1.8mg/d Z 2.8mg/d Z plus 9mg/d L 3.placebo	HFP	12 months
Weigert, G. et al.(2011)[33]	126 AMD patients (stages 2,3,and 4)	age 71.6±8.6 F66; M50	1.20mg/d/ L 2.placebo	Spectral fundus reflectance	6 months
Arnold, C. et al(2013)[34]	145 dry AMD patients	age 69.1±9.7 F79;M 66 BMI 27.8±4.34	1.10 mg L, 1 mg Z, 100 mg DHA, 30 mg EPA 2. 20 mg L, 2mg Z, 200 mg DHA, 60 mg EPA 3.placebo	1-wavelength reflection method	12 months
Garcia-Layana, A. et al.(2013)[35]	44 early AMD patients	F18; M26 age 68.53±8.43 BMI 25±1.45	1.12mgL 0.6mg Z 280mgDHA 2.placebo	HFP	12 months
Murray, I. J. et al(2013)[36]	72 AMD patients	age 70.5±8.7 F35; M37	1.10mg L 2.placebo	HFP	12 months
Huang, Y. M. et al.(2015)[37–39]	81 early AMD patients	F 47; M34 age 69.33±7.32 BMI24.67±3.22	1.10mgL 2.20mgL 3.placebo	Fundus autofluorescence	24 months
Azar, G. et al.(2017)[40]	126 AMD patients	F74; M52 age 75.3±7.61 BMI 25.69±4.68	1.5mgL1mgZ 2.placebo	Fundus autofluorescence	12months

Abbreviations: HFP, heterochromic flicker photometry; F, female; M, male; BMI, body mass index; Z: zeaxanthin; L: lutein.

### Effects of lutein supplementation in AMD patients

#### MPOD

**Fig 4:**
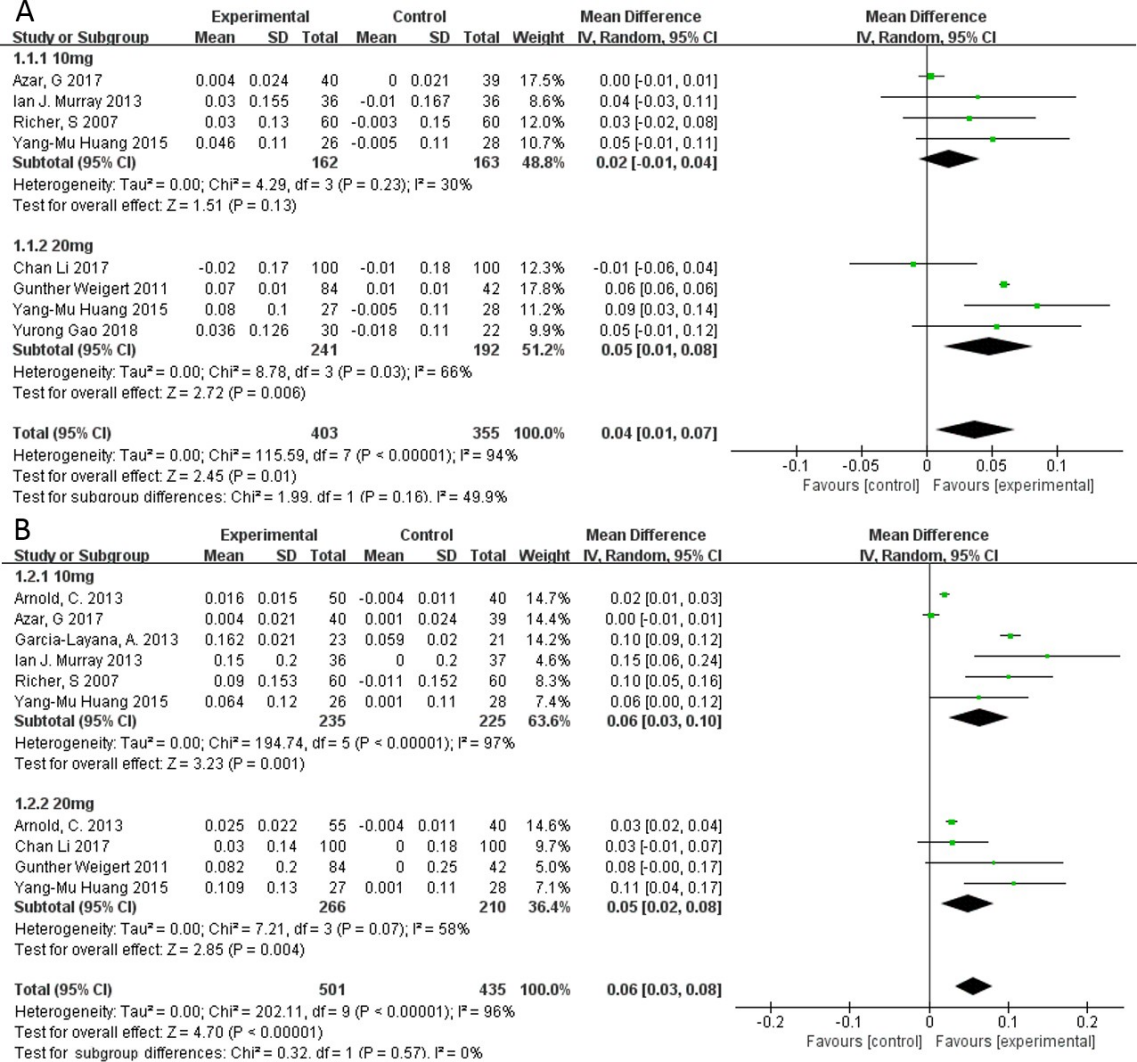
Forest plot showing the effect of lutein supplementation on macular pigment optical density in AMD patients when supplementation lasted (A) fewer than 6 months or (B) longer than 1 year.

Random-effects meta-analysis of all nine studies showed that lutein supplementation was associated with higher MPOD (MD 0.07; 95%CI 0.03 to 0.10). Stratified analyses based on length of supplementation showed that MPOD was significantly higher with 10 mg/d lutein than with placebo after more than 1 year (MD 0.06; 95%CI 0.03 to 0.10); but not after fewer than 6 months (MD 0.02; 95%CI -0.01 to 0.04). At the higher dose of 20 mg/d lutein, MPOD was significantly higher in the treatment group than the placebo group after fewer than 6 months (MD 0.05; 95%CI 0.01 to 0.08) as well as after more than 1 year (MD 0.05; 95%CI 0.02 to 0.08) (**Fig 4**).

#### Visual acuity

**Fig 5:**
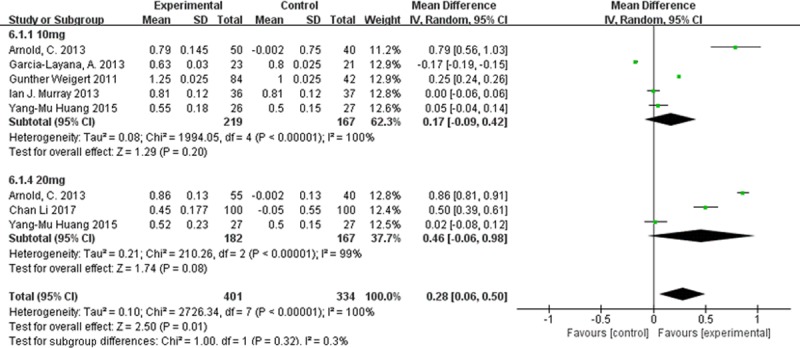
Forest plot showing the effect of lutein supplementation on visual acuity in AMD patients.

Random-effects meta-analysis of the six studies that included visual acuity as an outcome showed that lutein supplementation for longer than 1 year led to significantly greater visual acuity than placebo (MD 0.28; 95%CI 0.06 to 0.50, **Fig 5**).

#### Serum lutein concentration

**Fig 6:**
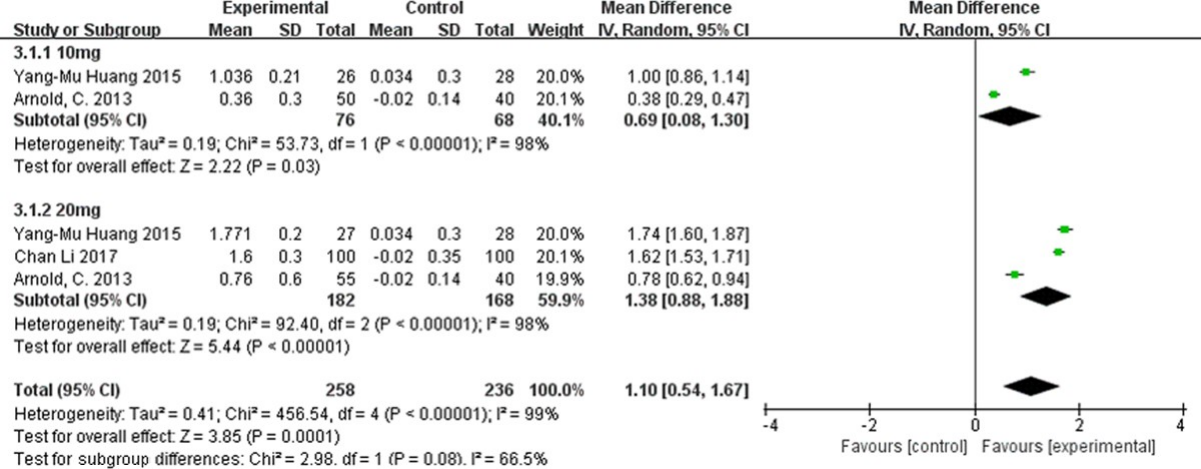
Forest plot showing the effect of lutein supplementation on serum lutein concentration in AMD patients.

Random-effects meta-analysis of the three studies that reported lutein concentrations in serum showed that lutein concentration was significantly higher in the treatment group than in the placebo group (MD 1.10; 95%CI 0.54 to 1.67, **Fig 6**).

#### Contrast sensitivity

**Fig 7:**
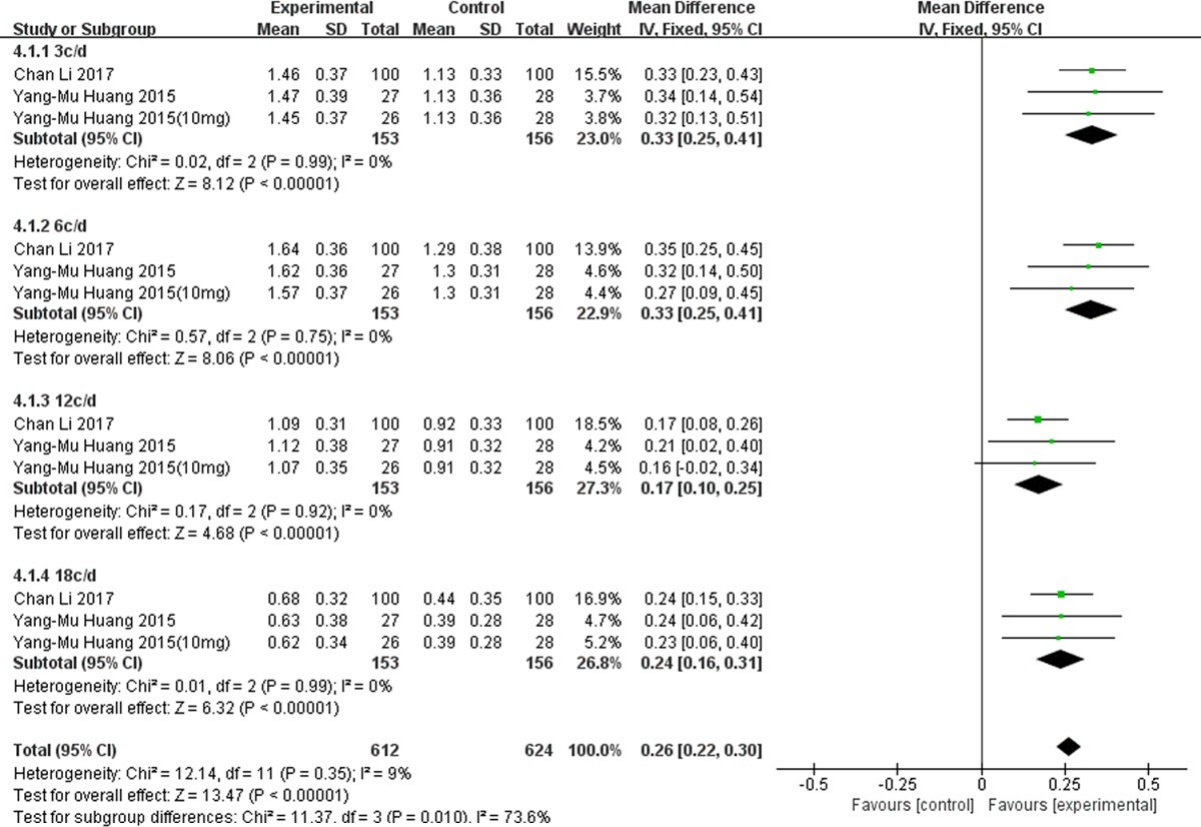
Forest plot showing the effect of lutein supplementation on contrast sensitivity in AMD patients.

Fixed-effects meta-analysis of the two studies that reported contrast sensitivity showed that lutein supplementation was associated with higher contrast sensitivity at spatial frequencies of 3, 6, 12 and 18 cycles per degree (MD 0.26; 95%CI 0.22 to 0.30, **Fig 7**).

## Discussion

Macular pigments, including the carotenoids lutein, play important roles in maintaining macular function and overall retinal function [4]. Lutein protects the retina by filtering out blue light and acting as an antioxidant [2, 41], and administering it to animals can significantly increase anti-oxidant enzyme levels and total anti-oxidant capacity [42]. Lutein and other carotenoids also suppress production of inflammatory mediators and reactive oxygen species, and they down-regulate NADPH oxidase subunit Nox4 [43, 44]. In cell culture studies, lutein protected ARPE-19 retinal cells from oxidative stress-induced cellular senescence [45], likely by quenching singlet oxygen [46]. These anti-oxidative properties have led to the suggestion that lutein may slow AMD progression and alleviate symptoms [47]. Our meta-analysis of randomized controlled trials suggests that this may indeed be the case. However, studies performed supplementation for a maximum of 5 years, leaving open the question of whether lutein supplementation can effectively treat AMD, a chronic progressive disease, in longer term. But due to the limitations of existing RCT trials, we can only evaluate the effect of lutein on AMD within 1 year.

Culture, animal and human studies have shown that retinal concentrations of lutein are inversely related to the risk of AMD [42, 45, 48]. The same study also provided evidence that low concentration of lutein may actually cause AMD [48]. Another study showed that low macular pigment level was related to worse vision in both healthy subjects and AMD patients [13]. Consistent with these findings, lutein supplementation improved vision in healthy subjects and reduced their risk of developing AMD [8, 49]. In addition, many clinical studies have shown that a diet that increases macular pigment levels can improve retinal function, especially in AMD patients with low MPOD [13, 50, 51].

In this way, our results that lutein supplementation can increase MPOD and visual acuity are in agreement with previous studies and a meta-analysis that included fewer studies than ours [52]. On the other hand, one meta-analysis suggested little to no benefit of lutein against AMD progression [53]. The results of that analysis may be less reliable because among the 19 studies included, only 6 examined lutein in AMD, and most data came from a single study (AREDS2). In addition, the meta-analysis did not stratify data by duration of supplementation, which ranged from six months to five years. As we know, AMD is a chronic progressive disease, intervention and follow-up time may affect the outcome effects.

The lack of efficacy for lutein supplementation in AMD patients may reflect the treatment that was too short and/or doses that were too low [23]. Our stratified analyses suggest the importance of providing lutein at sufficiently high doses and durations: daily doses of at least 20mg showed efficacy within 6 months, compared to 1 year for daily doses of 10mg. However, at least one study showed that 10 mg lutein had the same effect as 20 mg per day [52], which contradicts our results. This raises the possibility that the efficacy of lutein supplementation depends on multiple factors. In any event, lutein or zeaxanthin appears to be safe in rats at up to 400mg/kg per day [54], and lutein appears to be safe in humans at up to 20mg per day [55].

Another reason that clinical lutein studies have come to divergent results may be differences in the study populations. MPOD and the ability of response to carotenoid supplementation may differ with genetic variations [56, 57]. Unfortunately we could not assess the potential contribution of genetic polymorphism to our results because none of the studies in our meta-analysis included genotyping data. Some studies did record BMI, because BMI would affect the concentration of lutein in serum [58], which was found not to differ significantly between patients who received supplementation or not. One study suggested that iris color affects MPOD, but another showed no such relationship [59]. Iris color was not recorded in studies included in this meta-analysis and therefore could not be analyzed.

Another source of variability among studies in the literature is the different ways to determine MPOD. There are many different methods to measure MPOD, including fundus reflectance, auto-reflectance and psychophysical methods [60, 61]. Most studies in our meta-analysis used a heterochromatic flicker photometer, which is commonly used but not always reproducible in clinical [62]. Some of the included studies did not report how MOPD was measured. To minimize the impact of different MPOD measurement methods, our meta-analysis was based on the change in MPOD from before supplementation, rather than absolute MPOD after supplementation.

One weakness of our meta-analysis is that heterogeneity was relatively high. This may reflect differences in the type of supplementation: five of the studies involved supplementation with lutein in combination with other antioxidants, such as zeaxanthin, meso-zeaxanthin, and zinc. Such mixtures may be more effective at quenching singlet oxygen than individual carotenoids at the same concentration [63, 64]. Another source of heterogeneity is the type and stage of AMD: most patients in this meta-analysis had dry-type AMD, but one study included patients with wet-type disease. AMD stage varied across studies. Our finding should be verified and extended in studies that control for these factors.

## Conclusions

The available evidence suggests that dietary intake of lutein (10 or 20 mg/day) for more than 6 months can significantly improve MPOD and visual acuity in AMD patients.

## Supporting information

S1 File Table PRISMA 2009 checklist. (DOC)

S2 File PRISMA 2009 flow diagram. (DOC)
