# Supplementary material for: Effects of lutein supplementation in age-related macular degeneration
Source: PLoS One. 2019 Dec 30;14(12):e0227048. doi: 10.1371/journal.pone.0227048 (PMC6936877; doi:10.1371/journal.pone.0227048)
Supplement: S2 File — (DOC) [file pone.0227048.s002.doc]

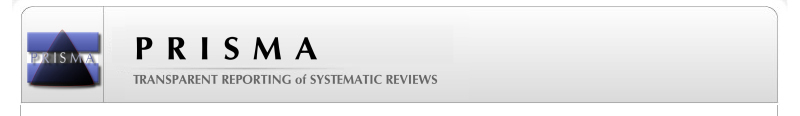
**PRISMA 2009 Flow Diagram**

**Screening**

**Included**

**Eligibility**

**Identification**

Records identified through 6 databases searching
(n = 288 )

Records after duplicates removed
(n = 194 )

Records screened
(n = 194 )

Records excluded
(n = 129 )

1. Observational studies (n=16)

2. Other topics (n=72)

3. Meeting abstracts (n=21)

4. Review and meta-analysis (n=17)

5. Animal research (n=1)

Full-text articles assessed for eligibility
(n = 65 )

Full-text articles excluded, with reasons
(n = 53 )

1. Non-RCT(n=32)

2. The outcome no MPOD(n=19)

3. Other languages(n=2)

Studies eligible for inclusion
(n = 12 )

Studies included (meta-analysis)
(n = 9 )

Same research group in different articles (n=3)
